# 3D bone shape from CT-scans provides an objective measure of osteoarthritis severity: Data from the IMI-APPROACH study

**DOI:** 10.1016/j.ostima.2024.100250

**Published:** 2024-09-14

**Authors:** James M Burlison, Michael A Bowes, Philip G Conaghan, Alan D Brett

**Affiliations:** a Imorphics Ltd, Worthington House, Towers Business Park, Wilmslow Road, Manchester M20 2HJ, UK; b Leeds Institute of Rheumatic and Musculoskeletal Medicine, University of Leeds, and NIHR Leeds Biomedical Research Centre, University of Leeds School of Medicine, Worsley Building, University of Leeds, Woodhouse, Leeds LS2 9JT, UK

**Keywords:** Machine vision, Osteoarthritis, Knee, Bone shape, Quantitative biomarkers

## Abstract

Decisions regarding total knee arthroplasty are usually made using a patient's own assessment of pain and the structural disposition of the joint as seen on plain film radiographs. Pain severity can fluctuate, and radiographs can be misleading, with the apparent joint status affected by anatomical orientation. An important component of the surgical management of knee osteoarthritis (OA) is the timing of surgical intervention: knee arthroplasty performed too early in the course of the disease may increase the need for revision surgery.

Femoral 3D bone shape (B-score) from MR images is an objective measure of OA severity and has been correlated with current and future risk of pain. We aimed to derive the B-score from CT images and compare it against the B-score derived from MR images.

We used baseline and 24-month image data from the IMI-APPROACH 2-year prospective cohort study, comprising pairs of CT and MR images taken for each subject-timepoint. The femur was automatically segmented in both CT and MR modalities using an active appearance model, a machine-learning method, to measure the B-score. Linear regression was used to test for correlation between measures. Limits of agreement and bias were tested using Bland-Altman analysis.

CT-MR pairs of the same knee were available from 424 participants (78 % women). B-scores from CT and MR were strongly correlated (CCC = 0.980) with negligible bias of 0.0106 (95 % CI: −0. 0281, +0.0493).

The strong correlation and small B-score bias suggests that B-scores may be measured reliably using CT images. Since CT images are used in planning robot-assisted knee arthroplasty, with further study B-scores derived from CT surgical planning images could in principle provide a useful objective input to deciding the appropriateness, timing and type of knee arthroplasty.

## 1 Introduction

Osteoarthritis (OA) of the knee is a slowly progressive disorder involving multiple joint tissue pathologies and causing substantial pain and disability. Knee arthroplasty is usually considered medically necessary when there is both pain or functional disability despite conservative therapies, and radiological evidence of joint space narrowing (JSN), a surrogate for cartilage loss. The assessment of structural pathology may also be made radiologically using grading systems such as Kellgren-Lawrence (KL) which includes definite JSN in grades 3 and 4 [1]. Radiographic determination of OA structural status and JSN, however, is imprecise due to its dependence on acquisition method and reader reliability [2].

It is recognized that an important component of the surgical management of knee OA is the timing of intervention. Knee arthroplasty performed too early in the course of the disease can mean a lack of benefit, and revision surgery (which is often complex) may be required at an earlier age when knee replacement is performed on a younger patient [3]. Pain is the primary reason for surgery, but pain variability can make decisions about timing more challenging. Pain due to knee OA demonstrates considerable within-person variability [4,5]. An objective and accurate quantitative measure of structural OA could provide useful information to aid the decision to perform knee arthroplasty.

Supervised learning of the 3D shape and appearance of the bones of the knee with an appropriate clinical imaging-derived model, can provide automatic segmentation of the anatomy in a consistent and repeatable fashion [6,7]. Such 3D methods have been shown to be superior to simpler 2D approaches [8]. Active Appearance Models (AAMs) comprise one such class of models and are a well-validated machine-learning method used to segment images to sub-voxel accuracy [[8], [9], [10]], producing a set of dense, corresponded landmarks delineating the surface of the segmented anatomy [6]. From the Principal Component Analysis (PCA) inherent to AAMs, it is possible to distil the segmented anatomical surface into a set of shape vectors [11]. This allows compact representation and direct comparison of the segmented anatomy between individuals and across populations [6].

Previous work using AAMs has successfully captured 3D shape features characteristic of knee OA, namely broadening and flattening of articulating bone surfaces and osteophytic ridge formation around their borders, as well as predicting radiographic OA onset and progression [6,8] and discriminating OA knees from healthy knees [6,9]. In the case of knee OA, changes in the shape of the distal femur were shown to have the greatest discrimination and responsiveness, prompting femoral bone shape to be adopted as an image-based metric of the disease state [6]. Distal femoral bone shape (“B-score”), automatically derived from 3D bone surfaces on magnetic resonance (MR) imaging [6], is employed as a quantitative imaging endpoint for OA disease status in Disease Modifying OA Drug (DMOAD) pharmaceutical clinical trials [[12], [13], [14]]. It comprises a unit scale defined in the femoral shape space, with its origin at the mean shape of the healthy subgroup (KL grades 0,1) of the Osteoarthritis Initiative (OAI) study cohort, oriented along the vector between this origin and the mean of the OA subgroup (KL grade > 2). Distance along the scale is normalized by the standard deviation of the non-OA subgroup [6]. As such, the B-score represents a statistical z-score which can be thought of as the number of healthy population standard deviations away from the mean healthy shape, along a line between healthy and OA. The B-score has been shown to be an objective, automated assessment of OA status with clinical risk defined for current pain and functional limitation, and 5-year risk of total knee replacement (TKR) [6]. The B-score has a typical range in an adult population of -2 to +8, with values from -2 to +2 roughly encompassing non-OA knees, and values from +2 to +8 comprising OA. Such negative B-score values are to be expected in healthy individuals, since the origin of the scale is defined as the mean healthy shape and, as such, the healthy population exhibits a spread on either side of this mean in the healthy-to-OA direction in femoral shape space. The smallest detectable difference of the B-score is 0.251 z-score units, giving roughly 40 distinguishable subdivisions for structural change [6]. As such, it represents a more sensitive, consistent and objective measure of OA state than radiographic systems such as KL grading.

Although the B-score was originally defined using MR imaging, computed tomographic (CT) imaging is more commonly used for 3D visualization of bony anatomy due to its high bone-soft tissue contrast and is routinely used in planning robot-assisted partial and total knee arthroplasty [15]. Previously we have determined that CT imaging may be employed to compute a B-score that is very similar to that derived from MR imaging of the same subject, with a small systematic bias [16]. In this study, we demonstrate an improved CT B-score algorithm that has negligible systematic bias and improved limits of agreement with the original MR B-score algorithm.

## 2 Methods

### 2.1 Data cohort and measurement of B-score

We used paired CT-MR image data from the IMI-APPROACH study, a European, 5-center, 2-year prospective cohort study. It includes clinical, imaging, biomechanical and biochemical parameters for a cohort of 297 participants (age 66.5 ± 7.1, women 230 (77 %), BMI 28.1 ± 5.3). Patients were stepwise selected for a high chance of structural and/or pain progression/sustained severity over two years [17]. The study was conducted in compliance with Good Clinical Practice (GCP), the Declaration of Helsinki, and the applicable ethical and legal regulatory requirements (for all countries involved) and was registered under clinicaltrials.gov identifier: NCT03883568. All participants received oral and written information and provided written informed consent.

**Fig. 1:**
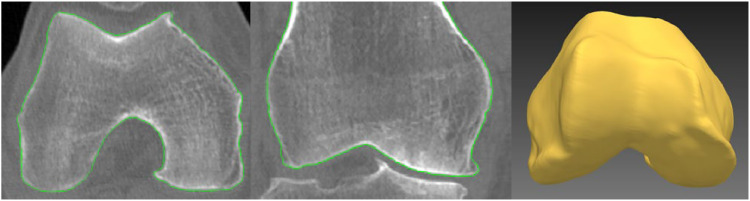
Example of 3D auto segmentation of the distal femur in a CT image from the IMI-APPROACH dataset. Left: transverse plane, middle: coronal plane, and right: 3D rendered surface.

The femoral surface was automatically segmented from CT and MR images using AAMs. Each imaging modality had its own separate AAM trained to segment that type of image with refinement of the CT model search result, similar to previous work [16]. An example of segmentation using the CT AAM model and refinement is shown in Fig. 1.

**Fig. 2:**
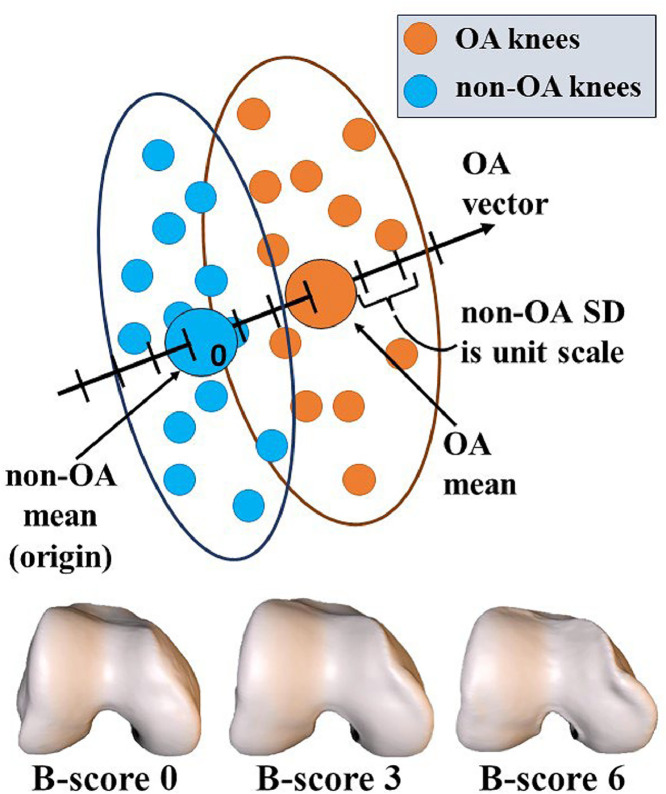
Schematic diagram of B-score definition and 3D renderings of bone shape changes with increasing B-score. (Top) Schematic diagram showing a lower-dimensional analogy of the B-score definition. Mean parameterized distal femur shapes are calculated for OA (KL2+) and non-OA (KL0) populations of the OAI [6] dataset. From this a vector in parameterized femoral shape space is defined from the non-OA population mean shape to the OA population mean shape. Units along this vector are normalized by the standard deviation of the non-OA population femur shape. To determine the B-score of an individual patient, the parameterized shape obtained from the corresponded auto-segmentation of their femur is projected onto this non-OA to OA shape vector and normalized by the non-OA femoral shape standard deviation. (Bottom) As the measured B-score increases, we see progressive anatomical signs of knee OA. Articulating surfaces broaden and flatten and osteophytic ridges form around their borders.

In either image modality, parameterized bone surfaces created from the femoral segmentation were projected onto a vector representing the direction of change of 3D MR bone shape from non-OA (consistently KL0 over 4 years) to OA (consistently KL≥2 over 4 years) knees in the OAI knee population [6]. The origin of the vector is the mean of the non-OA OAI population knee shape and one unit along the vector is one standard deviation of the distribution of projections of these non-OA knees onto this vector (Fig. 2). The resultant z-score comprises the B-score, which quantifies the normalized position of the knee surface projection onto this vector between population means. Moving along the population vector in the direction of increasing B-scores, we see many global anatomical changes in the distal femur consistent with OA progression, such as broadening and flattening of articular surfaces and osteophytic ridge formation and growth (Fig. 2).

**Fig. 3:**
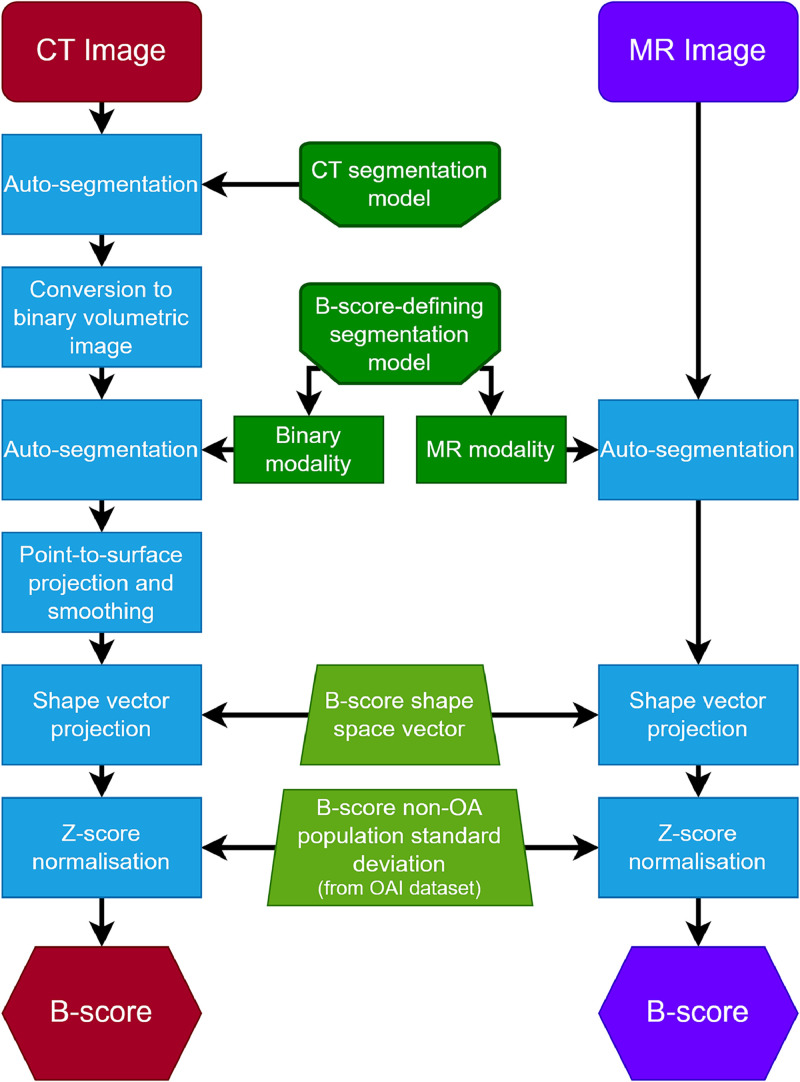
Flowchart illustrating the CT and MR derived B-score processes. The right side shows the MR B-score pipeline: flowing from the auto-segmentation of the input MR image to its shape-space projection onto the OAI B-score-defining shape vector (with Z-score normalization) to calculate the B-score. The left side shows the CT B-score pipeline: flowing from the auto-segmentation of the input CT image to the conversion of the segmented surface to a volumetric binary image and its subsequent segmentation with a binarized version of the B-score-defining AAM, followed by point-to-surface projection and smoothing. Following this, the resultant surface undergoes the same shape-space projection and normalization as in the MR pipeline, producing the B-score.

For MR images, the shape parameterization comes directly out of the AAM segmentation, since the femur model used to segment the OAI dataset in the definition of B-score is used to segment the MR images. This B-score defining model was built from DESSwe images of 96 participants (43 men, 53 women) from the OAI 0.B.1 group. The KL grade distribution of this training set comprised 43 KL0 and KL1, 7 KL2, 28 KL3 and 18 KL4 participants [6]. In the case of CT images, the refined segmentation model does not share the same shape parameterization as the MR model. This is because the shape parameterization depends on the set of corresponded shape model vertices that define the model surface. The MR and CT models use different arrangements of vertices. Therefore, the CT segmentation is first converted to a binary volumetric image. This image is then segmented by a binarized version of the MR AAM search model and then re-projected onto the original CT segmentation, with some small subsequent surface smoothing to mitigate any minor projective anomalies. This technique allows for re-parameterization in terms of the B-score-defining model, allowing for B-score measurements agnostic to modality and image segmentation model. The key algorithm step that improved upon versus earlier work [16] was the segmentation of the binary volumetric image; the segmentation behavior is now more consistent, with the final segmented surface fully optimized to convergence. In that previous work, the algorithm would sometimes produce a less optimized surface than this improved algorithm can produce. An overview of the CT and MR B-score algorithms is shown in Fig. 3.

### 2.2 Analysis of agreement between CT and MR B-score

Linear regression was used to test for B-score correlation between image modalities, with Pearson's correlation coefficient, r, Lin's Concordance Correlation Coefficient, CCC, and the linear regression coefficient of determination, R^2^, calculated as metrics of the correlation. Limits of agreement and bias were tested using Bland-Altman analysis. Images acquired from the same subject at baseline or 24-month timepoints were treated as independent. In the IMI-APPROACH dataset patient KL grade at baseline was also collected alongside CT/MR image data. To investigate the relationship between CT-derived B-score and KL grade, the baseline portion of this dataset was stratified by KL grade and the distribution of B-scores for each baseline KL grade cohort was determined.

## 3 Results

**Fig. 4:**
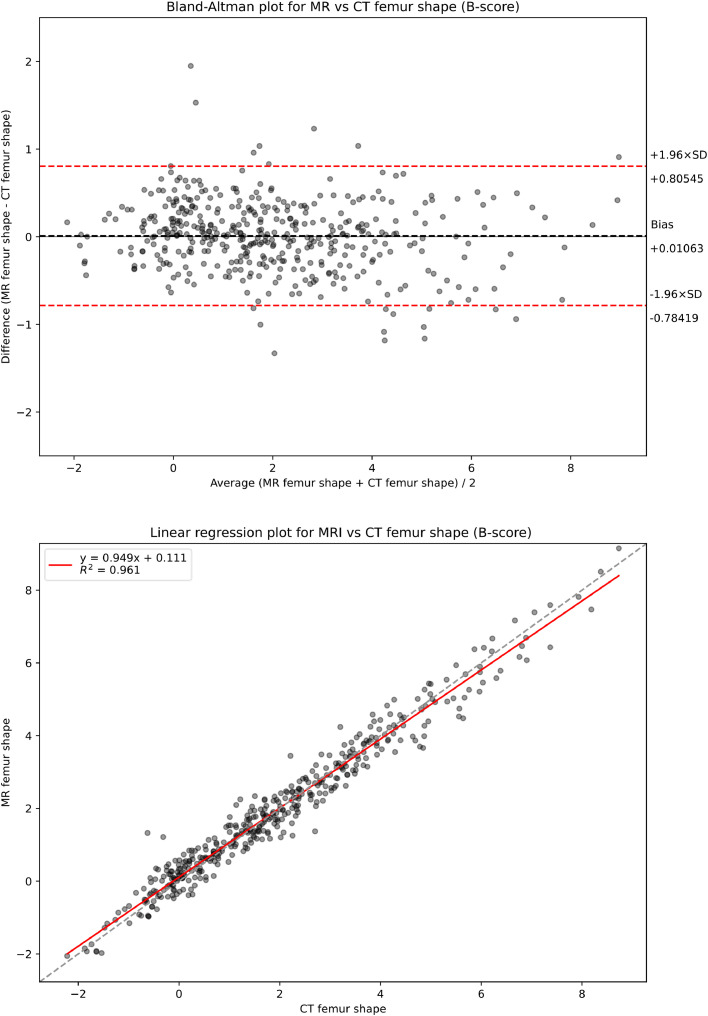
Bland-Altman and linear regression analyses of corresponding MR and CT B-scores. (Top) Bland-Altman plot comparing MR vs CT B-score measured from the same patient at the same timepoint pairs of CT and MR images. We see negligible MR-CT bias and a 95 % confidence interval limit of agreement of less than a single B-score unit, with no evidence of measurement value dependence in the bias. (Bottom) Linear regression fit of MR vs CT B-scores measured from the same patient at the same timepoint pairs of CT and MR images. Data shows a consistent strong linear correlation across a wide range of measured B-scores with a linear best-fit close to the dashed unity line.

There were 227 baseline and 202 24-month CT-MR image pairs of the same knee available, for a total of 429 CT-MR image pairs. After excluding cases where the image search algorithm failed, the final number of image pairs suitable for analysis was 424; with the exception of one CT image, which produced a poor-quality search result, the failures were all caused by the anatomy being partially outside the scan field of view. In the analysis set, there were 335 female knees (78 %). KL grading for the analyzed knee in these participants was KL0 19 %, KL1 31 %, KL2 30 %, KL3 16 % and KL4 3 %, with 5 knees ungraded in the original study data (1 %), providing coverage across the OA spectrum. The mean time between CT and MR image acquisition was 11 days (median: 0 days; 95th percentile: 107 days). Of the MR-CT scans 62% were acquired on the same day, and 99 % were acquired within 4 months of each other, a period of time which is unlikely to result in measurable anatomical change. To verify this, the analyses in Fig. 4 were repeated for the sub-cohorts of MR-CT scans acquired on the same day vs those acquired on different days. No meaningful differences were observed between them. The distribution of MR-CT B-score differences versus days between acquisition was also analyzed, with no evidence that the time between acquisitions had any effect on B-scores. Results of these analyses can be found in the supplementary material.

B-scores measured using CT and MR images were strongly correlated (Pearson's *r* = 0.980, linear regression coefficient of determination of linear model fit, R^2^ = 0.961 (Fig. 4, bottom) and Lin's CCC = 0.980) and showed good agreement, with a very small MR-positive bias of 0.0106 (95 % CI: − 0. 0281, +0.0493). Limits of agreement for B-score were − 0.784 and + 0.805. The Bland-Altman plot showed no apparent association between measurement values and bias (Fig. 4, top).

**Fig. 5:**
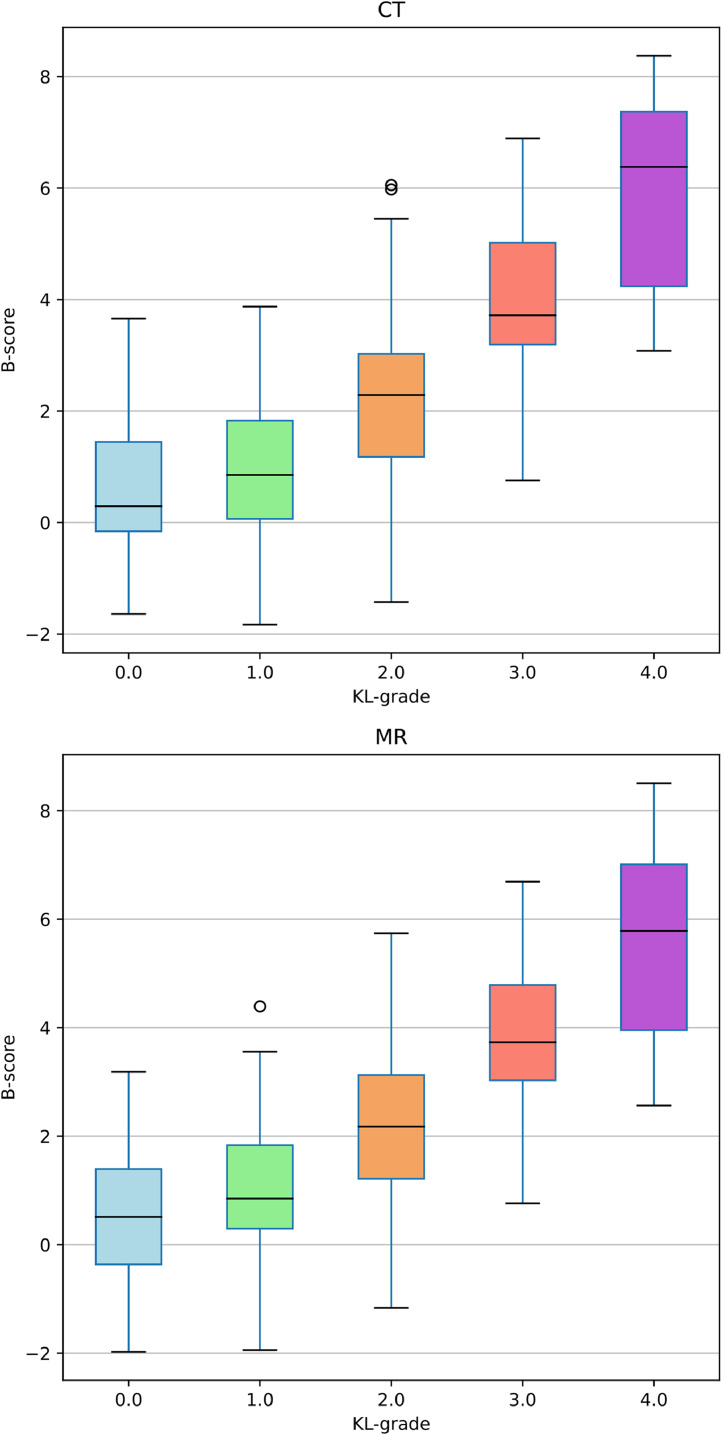
CT and MR derived B-score box and whisker plots for each KL grade. The horizontal line inside each box is the median. The boxes indicate the interquartile range (IQR). Whiskers mark the farthest data points lying above and below the box, within 1.5 times the IQR from it. The fliers (circles) indicate any data lying outside the whiskers. For both modalities, KL0 and KL1 overlap significantly whereas KL2+ show marked increase in both median B-score and inter-KL grade cohort separation of median B-score. The IQR for the KL4 cohort is markedly larger than the other cohorts possibly due in part to the larger variability in highly diseased knee anatomy, but most likely as a result of the small number of patient-timepoints (12) present in the IMI-APPROACH dataset with this highly advanced category of OA. The 5 ungraded knees were excluded from this analysis since without a KL grade they cannot be assigned a KL cohort.

The plots in Fig. 5 show a curvilinear relationship between both CT and MR-derived B-scores and KL grades with a marked upward movement in B-scores for KL grades of 2 and higher, which qualitatively agrees with the canonical KL grade boundary for radiographic OA by the identification of definite osteophytes. The interquartile range of the KL4 cohort is markedly broader than the lower KL grades. This may be partly due to the larger variability in the highly diseased knee anatomy of such advanced OA, but is also likely strongly affected by the small sample size of participants with such severe disease in the IMI-APPROACH study (*n* = 12 patient-timepoints for the KL4 cohort), which reduces the stability and accuracy of the sample interquartile range as an estimate of the true dispersion of the KL4 population.

## 4 Discussion

The strong correlation and close-to-zero B-score bias indicates that B-scores can be measured reliably using CT scans. The improvement in agreement metrics between MR and CT B-scores confirms that the improved CT B-score algorithm agrees more closely with MR B-scores than in earlier work (previously R^2^ = 0.938, CCC = 0.967, bias = 0.100 [95 % CI: 0.052, 0.14], Bland-Altman limits of agreement: − 0.896, + 1.096) [16]. Although it is likely that the bone surfaces identified using MR and CT will be at slightly different positions within the bone-cartilage boundary, the use of shape as opposed to geometric measurement as a metric here would appear to mean that this does not alter the B-score measurement of a knee.

While the radiographic KL grade is often used to stratify the knee joint by OA severity, it has several previously documented problems [18,19]. The scoring is based on 2D plane radiographs and can be reader-dependent and therefore subjective. The use of a 2D imaging modality means that a very small change in knee orientation can alter the determination of joint space narrowing and thereby alter the KL grade. In addition, the grades are more categorical than ordinal, with new OA-related features being introduced at each grade, which makes KL grades non-linear. Often, a KL grading of 3 or above is used when deciding the appropriateness of TKR surgery. We would expect the B-score to be a more reliable objective indicator of structural pathology when used for this purpose.

A systematic review of UK surgical registries and clinical studies showed that 85 %–90 % of TKRs lasted 15–20 years [20]. Thus, the younger the patient the more likely they will eventually require revision surgery. Arthroplasty performed too soon in the course of the disease can mean a lack of benefit [3], as TKR revision surgery is associated with poorer patient-reported outcomes, and TKR revision patients suffer significantly more disability and less improvement than those undergoing primary TKR [21]. If measures such as B-score were used to make a more objective and fine-grained determination of knee OA status during the assessment of TKR suitability, it is possible that the surgery could be performed later in life, reducing the chance of needing a revision, or that an alternative more conservative surgical approach such as partial knee replacement (PKR) could be chosen. For example, a patient presenting with a lower B-score, in combination with other clinically relevant indications, might be a better candidate for more conservative therapy than a patient with end-stage knee OA and a much higher B-score.

In the evolving field of robotic surgery, the use of CT is becoming routine for surgical planning. B-scores from CT could therefore represent an important imaging biomarker, providing critical objective insight into the disease state. Because it is constructed on a metric line from zero representing normal (KL0) knee shapes to higher scores for KL ≥ 2 knee shapes, B-scores provide an automated high-precision objective 3D version of what KL grades capture in 2D, but with much more informative content. In the future, it may be possible to use B-scores together with other important clinical and imaging information as part of a machine learning algorithm to predict long term post-operative patient outcomes for different interventions, such as PKR vs TKR.

This study had some limitations. A larger KL4 cohort size would have been useful, since this grade is often well-represented in a population of TKR candidates, but under-represented, as in this study, within clinical trials and observational OA studies. However, the participants with a higher B-score (including those with KL4 which are primarily in the B-score range 4–7) do not appear to have a different pattern than the rest of the study population in the correlation and limits of agreement visualizations. There was also no test-retest repositioning image data for CT available, which meant that we could not determine the smallest detectable difference of CT B-scores as a repeatability measure. By treating baseline and follow-up subjects as independent, there is the potential of underestimating the size of confidence intervals on certain kinds of measurements [22]. In this study however, we are comparing the agreement between and noise properties of two different techniques (CT and MR imaging) for making the same measurement (B-score), rather than comparing cross-sectional or longitudinal measurements between participants or timepoints. As such our analysis of B-scores does not estimate or report between-participant or within-participant variance.

B-scores are constructed using the OAI dataset separated into OA/non-OA knees by KL grade, and therefore have some properties of the KL grade and capture osteophyte growth and bone broadening and flattening, usually referred to as “attrition”. However, the B-score differs in that it is linear, continuous, automated (and therefore objective) and much more precise, with a resolution of around 40 discernible units between non-OA and severely OA knees [6]. Although joint space narrowing is to some extent incorporated in the B-score, through its construction using KL grades to define OA/non-OA OAI populations, a more accurate determination of JSW using a 3D measure from CT images might also be combined with B-score values to provide additional information on cartilage integrity and/or meniscal competence for surgical decision-making.

In summary, we have shown that B-scores can be reliably derived from CT imaging and show negligible bias, with no discernible measured value dependence, compared to MR-derived B-scores. This paves the way for future use of B-scores as a knee OA imaging biomarker derived from CT images as well as MR.

## Disclosure of interests

JMB, ADB and MAB are employees of and shareholders in Stryker. PGC has participated in speakers bureaus or consultancies for: AbbVie, Diffusion, Eli Lilly, Galapagos, Genascence, GSK, Grunenthal, Janssen, Levicept, Novartis, Pacira, Stryker, Takeda.

## Declaration of competing interest

The authors declare the following financial interests/personal relationships which may be considered as potential competing interests:

Philip G Conaghan reports financial support was provided by EU EFPIA Innovative Medicines Initiative Joint Undertaking. James M Burlison reports a relationship with Stryker Corporation that includes: employment and equity or stocks. Alan D Brett reports a relationship with Stryker Corporation that includes: employment and equity or stocks. Michael A Bowes reports a relationship with Stryker Corporation that includes: employment and equity or stocks. Philip G Conaghan reports a relationship with AbbVie Inc that includes: consulting or advisory and speaking and lecture fees. Philip G Conaghan reports a relationship with Diffusion Pharmaceuticals Inc that includes: consulting or advisory and speaking and lecture fees. Philip G Conaghan reports a relationship with Eli Lilly that includes: consulting or advisory and speaking and lecture fees. Philip G Conaghan reports a relationship with Galapagos that includes: consulting or advisory and speaking and lecture fees. Philip G Conaghan reports a relationship with Genascence that includes: consulting or advisory and speaking and lecture fees. Philip G Conaghan reports a relationship with GSK that includes: consulting or advisory and speaking and lecture fees. Philip G Conaghan reports a relationship with Grünenthal Ltd that includes: consulting or advisory and speaking and lecture fees. Philip G Conaghan reports a relationship with Janssen Pharmaceuticals Inc that includes: consulting or advisory and speaking and lecture fees. Philip G Conaghan reports a relationship with Levicept that includes: consulting or advisory and speaking and lecture fees. Philip G Conaghan reports a relationship with Novartis that includes: consulting or advisory and speaking and lecture fees. Philip G Conaghan reports a relationship with Pacira BioSciences Inc that includes: consulting or advisory and speaking and lecture fees. Philip G Conaghan reports a relationship with Stryker Corporation that includes: consulting or advisory and speaking and lecture fees. Philip G Conaghan reports a relationship with Takeda Pharmaceutical Company Limited that includes: consulting or advisory and speaking and lecture fees. If there are other authors, they declare that they have no known competing financial interests or personal relationships that could have appeared to influence the work reported in this paper.

## Appendix Supplementary materials

Image, application 1
