# Supplementary material for: 3D bone shape from CT-scans provides an objective measure of osteoarthritis severity: Data from the IMI-APPROACH study
Source: Osteoarthr Imaging. 2024 Sep 14;4(4):100250. doi: 10.1016/j.ostima.2024.100250 (PMC13228723; doi:10.1016/j.ostima.2024.100250)
Supplement: Supplementary file 1 [file mmc1.docx]

3D Bone Shape from CT-Scans Provides an Objective Measure of Osteoarthritis Severity: data from the IMI-APPROACH study

Supplementary Material


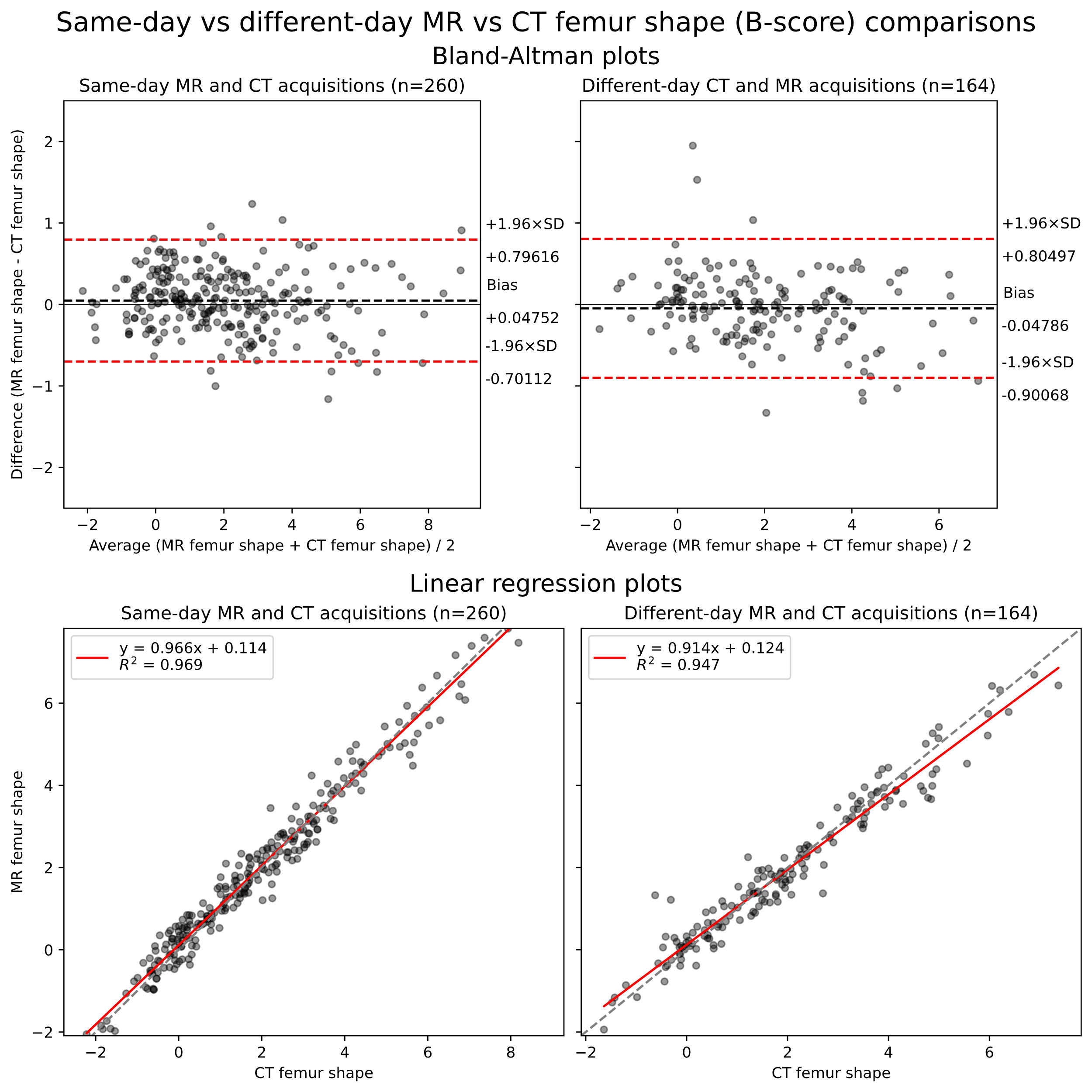


**Figure S1. Repeat of Bland-Altman and Linear regression analyses of corresponding MR and CT B-scores, split into same-day and different-day sub-cohorts.**(Top) Comparison of Bland-Altman plots of MR vs CT B-scores for MR-CT acquisitions on the same day (left) and different days (right). We see a minor difference in the 95% confidence interval LOAs and negligible difference in the bias.
(Bottom) Comparison of linear regression fits of MR vs CT B-scores for MR-CT acquisitions on the same day (left) and different days (right). We see a minor difference in the fitted gradient and negligible difference in R^2^. Overall, we see little evidence of any significant difference between the same-day CT-MR and different-day CT-MR sub-cohorts.

|  | **Same-day MR and CT acquisitions** | **Different-day MR and CT acquisitions** |
| --- | --- | --- |
| **Bland-Altman plots** | bias: +0.047521  95% CI on bias: [+0.000875: +0.094167]  lower loa: -0.701120 95%CI on loa: [-0.781912: -0.620327]  upper loa: +0.796161  95% CI on loa: [+0.715368: +0.876954] | bias: -0.047857  95% CI on bias: [-0.114948: +0.019235]  lower loa: -0.900682 95% CI on loa: [-1.016887: -0.784476]  upper loa: +0.804968  95% CI on loa: [+0.688763: +0.921174] |
| **Linear regression plots** | coefficient of determination (R-squared): 0.969  intercept: 0.114  slope: 0.966  mean squared error: 0.140 | coefficient of determination (R-squared): 0.947  intercept: 0.124  slope: 0.914  mean squared error: 0.162 |

**Table S1. Bland Altman and Linear regression parameters associated with analyses in Figure S1.**(Top row) Bland-Altman bias and limits of agreement, with associated 95% confidence intervals.
(Bottom row) Linear regression coefficient of determination, intercept, slope and mean squared error.


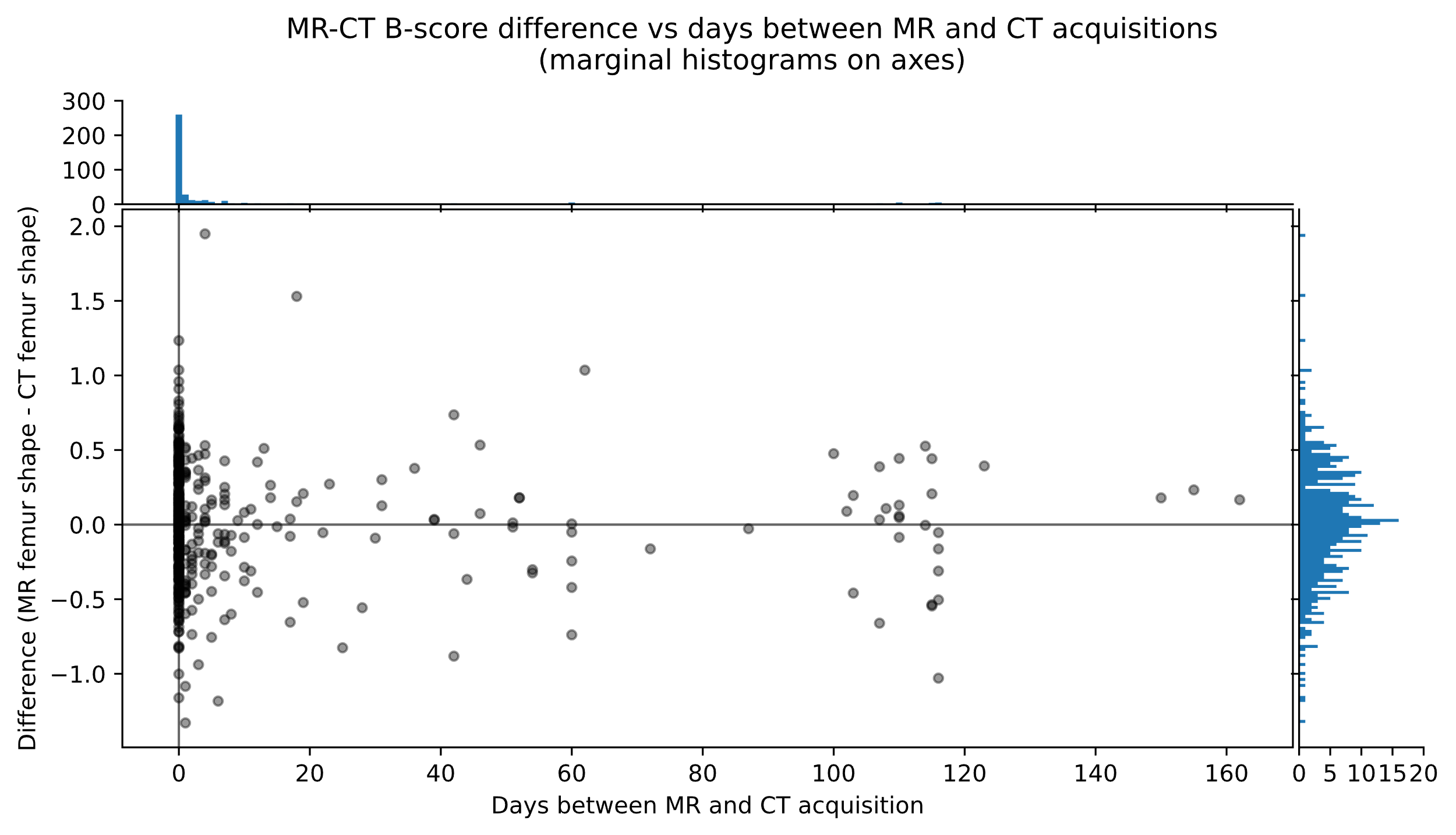


**Figure S2. Scatter plot and associated marginal histograms of MR - CT B-score difference vs number of days between the MR and CT acquisitions.**From the main scatter plot, assisted by the marginal histograms, we see both the preponderance of MR-CT image pairs with zero days between acquisitions, and the lack of evidence of any dependence of MR-CT B-score difference on the number of days between MR and CT acquisition.
